# Functional Derangements of the Liver

**Published:** 1874-09

**Authors:** Chas. Murchison


*THE LONDON LANCET—June*, 1874:

FUNCTIONAL DERANGEMENTS OF THE LIVER.—*By Chas. Murchison, M.D.*—* * * Professional opinion as to what constitutes functional disorder of the liver is vague and unsatisfactory. There is no expression more common among both patients and their doctors than that the "liver is out of order," or that certain symptoms are due to "biliousness," and yet few medical writers have undertaken to define with accuracy what symptoms are referable to a disordered liver. It is to be feared that symptoms are sometimes referred to the liver with which it has little or no concern; while, on the other hand, there are grounds for suspecting that many symptoms, at first sight apparently referable to other organs, and even grave degenerations of tissue and organic disease, not only of the liver itself, but throughout the body, may be traced back to functional derangements of the liver, although some of these may as yet be imperfectly understood. * * * Before preceeding to discuss the results of derangement of the liver, it will be necessary for me to refer to the functions of the organ in its healthy state. * * *

Although it was *a priori* improbable that the largest gland in the body, deriving large supplies of blood from different sources, as well as holding peculiar relations to the blood returning from the placenta in the fœtus and from the stomach and intestines in the adult, should have as its sole function the secretion of a fluid which is apparently of less importance in digestion than the gastric or pancreatic juice, yet for nearly two centuries the only object of the liver was believed to be the secretion of bile; and down to the present day its functional derangements are constantly spoken of as restricted to the secretion of bile abnormal in quantity. * * *

From what has been stated, it follows that the functions of the liver may be summed up under three heads, viz:

1. The formation of glycogen, which contributes to the maintenance of animal heat, and to the nutrition of the blood and tissues, and the development of white blood-corpuscles.

2. The destructive metamorphosis of albuminoid matter, and the formation of urea and other nitrogeneous products, which are subsequently eliminated by the kidneys, these chemical changes also contributing to the development of animal heat.

3. The secretion of bile, the greater part of which is reabsorbed, assisting in the assimilation of fat and peptones, and probably in those chemical changes which go on in the liver and portal circulation; while part is excrementitious, and, in passing along the bowel, stimulates peristalsis and arrests decomposition. * * *

The few medical writers who have described the functional derangements of the liver have arranged them under the three following heads: 1. Diminished secretion of bile; 2. Increased secretion of bile; 3. Secretion of morbid or altered bile. But this classification fails to recognize the most important functions of the liver; and, from what has been stated, it follows that the quantity and quality of the bile discharged from the bowel, upon which the classification is based, are no certain tests of the amount and quality of the bile secreted by the liver. The quantity secreted being the same, the quantity discharged from the bowel will vary with whatever stimulates or impedes absorption. Any substance like calomel or podophylin, or certain articles of diet, which irritates the commencement of the small intestines, will sweep along the bile before there is time for absorption, and thus cause an increased flow from the bowel, without the secretion from the liver being necessarily increased. Moreover, it must often be impossible to say whether the morbid or altered appearances of the bile in the fæces be due to a vitiated bile, or to changes which the bile has undergone in its passage through the bowel. For these reasons I have ventured to suggest another classification of the functional derangements of the liver, based upon what are now believed to be the normal functions of the gland, and upon the symptoms which a disordered liver may excite in the different physiological systems of the body.

*Classification of the Functional Derangements of the Liver.*

I. Abnormal Nutrition.
II. Abnormal Elimination.
III. Abnormal Disintegration.
IV. Derangements of the Organs of Digestion.
V. Derangements of the Nervous System.
VI. Derangements of the Organs of Circulation.
VII. Derangements of the Organs of Respiration.
VIII. Derangements of the Unrinary Organs.
IX. Abnormal Conditions of the Skin.

I. *Abnormal Nutrition.*—Functional derangements of the liver may lead directly to (1) an abnormal deposition of fat, or to (2)

the opposite condition of emaciation. Indirectly, also, the nutrition of the body may become seriously impaired from derangements of the disintegrative functions of the liver.

1. *Corpulence*, by which so many persons are inconvenienced, owes its origin to different causes. * * * Although we can only as yet speculate as to the precise nature of the morbid process, we know that, in animals eating much farinaceous, saccharine or oleaginous food, the proportion of fatty particles in the secreting cells of the liver is much larger than in animals moderately fed and taking much exercise. * * *

2. *Emaciation* may be induced by functional derangements of the liver in different ways.

(*a*) In consequence of a deficient formation of bile, or its impeded passage into the bowel, the assimilation of fatty and albuminous matters is interfered with. * * *

(*b*) But, secondly, emaciation may result from derangement of the glycogenetic function of the liver. *Diabetes*, in fact, may be said to be in most instances a functional derangement of the liver. It would be out of place here to consider in detail what are now believed to be the various causes of glycosuria; but, briefly, they may be said to come under one of the three following heads:

1. Imperfect glycogenesis in the liver.

2. An increased conversion of glycogen into sugar; the destruction of the sugar remaining unaltered.

3. Diminished destruction of sugar.

II. *Abnormal Elimination.*—The symptoms usually associated with a deficient excretion of bile are an irregular, usually costive, state of the bowels; the stools being insufficiently colored with bile, and of a pale-yellow, drab, or whitish color; loss of appetite; a white or yellowish furred tongue; a disagreeable, often bitter, taste in the mouth, especially in the morning; flatulence; a sallow or muddy tint of the skin; (indicating, unless there be concurrent hyperæmia of the liver, anæmia rather than jaundice,) dingy conjunctivæ; languor and disinclination for exertion; frontal headache; dulness and heaviness; drowsiness after meals; great depression of spirits, and sometimes hypochondriasis, and frequent deposits of lithates in the urine on cooling. These symptoms are very apt to be induced, especially towards middle life, by sedentary or indolent habits; the habitual use of rich or indigestible food; neglect of the bowels; great or protracted anxiety of the mind; or by a general want of vigor, consequent upon disease of the heart or some other organ, and the tendency to them is in many cases inherited. They are commonly, and perhaps correctly, ascribed to what is called "torpor of the liver;" but the non-excretion of bile may possibly be merely one of the symptoms, rather than the cause, of the morbid state; the real cause being the retention in the system, not of bile, but of those products of disintegration which it is the purpose of the kidneys to eliminate. At the same time, it is very probable that engorgement of the liver with bile interferes with the normal processes of disintegration of albumen which take place in the gland.

III. *Abnormal Disintegration.*—Modern investigations, pathological as well as physiological, go far to prove that one of the chief functional derangements of the liver, if it be not the foremost of all, is an imperfect disintegration of albuminous matter. * * * The most common of these changes in the urine are deposits on cooling of lithic acid, lithates, and pigmentary matters. * * * I need not remind an audience such as that which I have the honor to address that deposits in the urine of lithic acid or lithates are not due to any morbid condition of the kidneys. What I wish to insist upon is that the frequent occurrence of these deposits in the urine ought always to be regarded as a sign of functional derangement of the liver, arising from causes sometimes temporary, at other times more or less permanent. * * *

Abnormal disintegration of albuminous matter in the liver may lead to a morbid condition of the blood and of the entire system, which often manifests itself in lithuria. This morbid state of the blood I propose to designate lithæmia.

When oxidation is imperfectly performed in the liver, there is a production of insoluble lithic acid and lithates, instead of urea, which is the soluble product resulting from the last stage of oxidation of nitrogenous matter. * * *

Of the symptoms indicating this condition, the most common are:

A feeling of weight and fulness at the epigastrium and in the region of the liver.

Flatulent distension of the stomach and bowels.

Heart-burn and acid eructations.

A feeling of oppression and often weariness and aching pains in the limbs, or of insurmountable sleepiness after meals.

A furred tongue, which is often large and indented at the edges, and a clammy, bitter, or metallic taste in the mouth, especially in the morning.

Appetite often good; at other times anorexia and nausea.

An excessive secretion of viscid mucus in the fauces and at the back of the nose.

Constipation, the motion being scybalous; sometimes too dark, at others too light, or even clay-colored. Occasionally attacks of diarrhœa, alternating with constipation, especially if the patient be intemperate in the use of alcohol.

In some patients, attacks of palpitation of the heart, or irregularity or intermission of the pulse.

In many patients, occasional attacks of frontal headache.

In many patients, restlessness at night and bad dreams.

In some patients, attacks of vertigo, or dimness of sight, often induced by particular articles of diet. * * *

From what has been already stated, it is clear that the kidneys and liver are intimately connected in their functions; the main object of the kidneys being to eliminate certain products which are in great part secreted in the liver. Derangements of one organ are, therefore, very likely to lead to disorder of the other. In the first place, my experience has led me to regard lithæmia as one of the chief causes of "acute bright's disease," or acute nephritis.

